# Assessment of quality of life using the EORTC 30 protocol in patients with soft tissue sarcoma undergoing surgical treatment

**DOI:** 10.1590/0100-6991e-20243766-en

**Published:** 2024-11-07

**Authors:** EURICO CLETO RIBEIRO DE CAMPOS, ELDER DALAZOANA, PEDRO AFONSO KONO, MARCELO AUGUSTO DE SOUZA, NATÃ DE JESUS PROENÇA, MELQUESEDEQUE DOS SANTOS, MATHEUS VON JELITA SALINA, PEDRO SCHNAIDER ZANOLLA, LETÍCIA MIDORI MICHALAWISKI YAMAOKA, LUANA NAOMI MIYAWAKI

**Affiliations:** 1 - Universidade Estadual de Ponta Grossa, Departamento de Cirurgia Geral - Ponta Grossa - PR - Brasil; 2 - Faculdade Evangélica de Medicina Mackenzie do Paraná, Departamento de Oncologia - Curitiba - PR - Brasil

**Keywords:** Quality of Live, Oncology, Chemotherapy, Qualidade de Vida, Oncologia, Sarcomas de Partes Moles

## Abstract

**Objective:**

To evaluate the quality of life among patients with Soft Tissue Sarcomas treated at the Evangelic Mackenzie Hospital (HUEM) from 2018 to 2024 and undergoing surgical treatment.

**Material and Methods:**

descriptive and cross-sectional analysis of 23 patients with soft tissue neoplasia who underwent surgery and whether they underwent neoadjuvant or adjuvant clinical treatments. Epidemiological, clinical, and pathological data were considered. The EORTC 30 protocol was the instrument used for assessing the patients’ quality of life.

**Results:**

the main cases were located at extremity in females. Mean age at diagnosis was 47 years. All patients were symptomatic at diagnosis, with pain and a palpable mass being the most prevalent symptom and clinical sign, respectively. The mean time from the onset of symptoms to the date of diagnosis was 9 months. The mean tumor size was 11.68cm. Considering the functional scale, the items physical functioning, role performance and social function were the most affected in the assessment of quality of life. Fatigue and loss of appetite were the most common sign and symptom, respectively. A global measure of quality of life achieved high rates when 50% of patients rated as excellent.

**Conclusion:**

Continuous and multidisciplinary oncological care provided to the patient allows for better symptom control, resulting in higher quality of life, which positively impacts the patient’s adherence to treatment, their progression, and possibly their survival.

## RESUMO

**Objetivo:**

Avaliar a qualidade de vida de pacientes portadores de Sarcomas de Partes Moles tratados no Hospital Universitário Evangélico Mackenzie (HUEM) no período de 2018 a 2024 e submetidos à tratamento cirúrgico.

**Material e Métodos:**

análise descritiva e transversal de 23 pacientes portadores de neoplasia de partes moles operados e submetidos ou não a tratamentos clínicos neoadjuvantes ou adjuvantes. Foram considerados dados epidemiológicos, clínicos e patológicos. A avaliação da qualidade de vida dos pacientes foi realizada utilizando o protocolo EORTC 30.

**Resultados:**

Predominaram pacientes do sexo feminino e com sarcomas de partes moles de extremidades. A idade média ao diagnóstico foi de 47 anos. Todos eram sintomáticos ao diagnóstico, sendo a dor e massa palpável o sintoma e sinal clínico mais prevalente, respectivamente. O tempo médio desde o inicio dos sintomas até a data do diagnóstico, foi de 9 meses. O tamanho tumoral médio foi de 11,68 centímetros. Considerando a escala funcional, os itens: capacidade física, desempenho de papéis e função social foram os mais afetados na avaliação da qualidade de vida. Fadiga e perda de apetite foram os sinais e sintomas mais comuns, respectivamente. A medida global de qualidade de vida alcançou taxas elevadas demonstrando boa qualidade de vida dos pacientes.

**Conclusão:**

O cuidado oncológico contínuo e multidisciplinar oferecido ao paciente possibilita um melhor controle dos sintomas, levando a uma qualidade de vida mais elevada, o que influencia positivamente a adesão do paciente ao tratamento, sua evolução e, possivelmente, a sua sobrevida.

## INTRODUCTION

Cancer is the second leading cause of mortality in the Brazilian population, surpassed only by cardiovascular diseases. It is estimated that, within the next ten years, neoplasms will become the main cause of death. For the three-year period from 2023 to 2025, 720,000 new cancer records are expected^1^.

Histologically, soft tissue sarcomas (STS) correspond to malignant neoplasms originating from primitive mesenchymal cells that reproduce soft tissue components, except for bone tumors and neoplasms of hematolymphoietic origin. They are more common in the body extremities, and may also affect the retroperitoneum, head and neck, and trunk^2^.

The main therapeutic modalities are surgery, radiotherapy, and chemotherapy^3^. Surgery is the main treatment for soft tissue sarcomas, and these are extensive procedures with morbidity resulting from the resection of muscle and bone tissues, as well as extensive reconstructions of vascular and soft tissues^2^.

Until the 80s, survival rates were less than 40% and most surgical procedures were limb amputations, with most of these patients dying in the first year due to lung metastases. Advances in diagnostic methods, staging, chemotherapy protocols, and surgeries have allowed for an increase in survival rates and indication of more conservative surgeries, positively impacting patients’ quality of life^2^ ^,^ ^4^.

There are few studies investigating the quality of life in soft tissue sarcomas. Even with multiple technological advances and a better understanding of the molecular biology of neoplasms, with new treatments aimed at the present molecular alterations and lower toxicity rates, many of these treatments end up impacting patients’ quality of life temporarily or permanently, imposing work, social, and family restrictions^4^.

## GOALS

To assess the quality of life of patients with Soft Tissue Sarcomas undergoing surgical treatment with the EORTC QLQ 30 protocol, as well as to evaluate the main epidemiological, clinical, and pathological factors involved in the treatment of these patients.

## METHODS

We obtained epidemiological, clinical, and pathological data of the patients from the medical records present in the files of the Oncology Service of a University Hospital.

We included patients with soft tissue neoplasms duly confirmed by histological analysis and classified in clinical stages I to IV, who received treatment exclusively within the scope of this medical center. We excluded patients who received treatment at other oncology institutions during the disease, as well as those whose neoplasm was not histologically confirmed and those whose clinical records presented incomplete information.

We studied the quality-of-life levels with the EORTC 30 form, which has been translated into Portuguese in 2000. The same medical professional applied the questionnaire to all patients during the return after standard treatment: for operated patients, within 180 days postoperatively, and for those who required adjuvant treatment, 90 days after it ended. This questionnaire consists of 30 questions and several scales, which include functional skills (such as physical, cognitive, emotional, social function, and task performance), symptom scales (including pain, fatigue, nausea, and vomiting), as well as an assessment of symptoms (such as dyspnea, inappetence, insomnia, constipation, and diarrhea), along with an assessment of quality of life and global health^5, 6^. 

The median follow-up time of patients was 18.7 months. 

The anatomopathological reports were issued exclusively by a specific Pathological Anatomy service, and the ASA Karfoski and ECOG classifications were performed by a single oncologist^7^. 

### Statistical analysis

The follow-up period was calculated in months and corresponded to the time interval between the date of surgery and the date of the patient’s last clinical follow-up. As comparative tests were applied in the present study, we decided to use descriptive statistics to present the results of the study. For the statistical analysis, we used the SPSS (Statistical Package for Social Science) for Windows, version 16.0. Quantitative variables were expressed as means and standard deviations, and qualitative ones, as their absolute and relative frequencies.

To calculate the score of each scale, we used a raw score (RS), which represents the value of the answers in the questionnaire divided by the number of items, as exemplified by Paiva SM^6^. The final score of the scale was determined by the following formula: Final score = [(RS -1) / range] x 100, the range being the difference between the maximum and minimum values of the item analyzed.

### Ethical aspects

This research project was submitted to and approved by the Ethics in Research Committee of the Evangelical University Hospital of Curitiba, under number 6.304.489. All patients signed an informed consent form.

## RESULTS

Among the 23 patients analyzed, 13 (56%) were female and 10 (44%) were male. The mean age was 47 years, and the median was 46.5 years.

All patients were symptomatic at diagnosis. Pain was the most common and prevalent single symptom, in 28.33% of the patients. A palpable mass was the most common clinical sign, with a mean tumor size of 11.68 centimeters, ranging from 2.4 to 30.6 centimeters. The mean time since the onset of signs and symptoms was nine months, and the median, 10 months, a delay that occurred on the part of the patient and of the medical team. Soft tissue sarcoma neoplasms of the extremities predominated (65%). 

We analyzed performance status with the Eastern Cooperative Oncology Group (ECOG) score. Most patients, 55%, were categorized as ECOG 0. There were no patients classified as ECOG 3. The ASA (American Society of Anesthesiology) classification was also routinely determined, due to its easy application and prognostic value like that of the ECOG. The values observed for ASA 1, 2, and 3 were, respectively, eight (34%), 12 (52%), and three patients (14%).

All patients had locally advanced or metastatic neoplasms at diagnosis. Clinical stages I and II were present in 39% (nine) and 8.9% (two) of patients, respectively, whereas 11 patients (47%) and one patient (5.1%) were clinical stages III and IV, respectively. Sarcomas of high histological grade predominated. The mean follow-up time was 18.7 months. Of the patients with comorbidities (45%), hypertension and diabetes mellitus were the most frequent. Table 1 shows the descriptive statistics, clinical presentation, and follow-up time of the patients studied. 

**Table 1:** Characteristics of the 23 patients participating in the study.

Variables	Distribution
Demographic	
Age, years (median)	47(46.5)
Female	13(56%)
Male	10 (44%)
Clinical presentation	
TNM Staging (2018)	
Variables	Distribution
CS I	39%
CS II	8,9%
CS III	47%
CS IV	5,1%
Degree	
Low grade STS	20%
High grade STS	45%
Desmoid	30%
GIST	5%
Location	
Extremitiy	65%
No-extremity	35%
Symptoms	
Yes	100%
No	0
Associated diseases	
Yes	45%
No	55%
Follow-up	
Time, months (median)	18,7(17,1)

STS: soft tissue sarcoma; CS: clinical staging; GIST: gastrointestinal stromal tumor.

Table 2 shows the mean and standard deviation of the EORTC 30 questionnaire items.

**Table 2:** Results of the EORCT 30 quality of life measurement instrument.

Scale items	Mean(%)	Standard deviation
Functional Scale		
Physical function	26,66	33,33
Paper Performance	31,66	33,33
Emotional function	11,66	16,66
Cognitive function	20,38	8,66
Social function	24,16	33,33
Symptom scale		
Fatigue	18,33	16,66
Nausea/vomiting	2,5	8,33
Pain	28,33	8,33
Symptoms		
Dyspnea	3,33	16,66
Insomnia	11,66	33,33
Loss of Appetite	8,33	33,33
Constipation	10,0	33,33
Diarrhea	3,33	16,66
Financial difficulties	25,00	33,33
Global QoL	73,75	16,66

QoL: quality of life.

## DISCUSSION

Cancer is one of the main causes of death in Brazil, with an increasing incidence, according to a report by the National Cancer Institute^1^. Many patients will be diagnosed with neoplasms in advanced stages, which will result in more pronounced and debilitating symptoms, negatively impacting their quality of life, as well as more extensive and debilitating surgical procedures for patients.

Regardless of the response to neoadjuvant treatment, surgery will be used in the treatment of sarcomas and will sometimes consist of the removal of muscle groups, bone, blood vessels, lymphatics, and skin. Despite the clinical and technological advances in the management of STS, the toxicity and morbidity of the treatments employed can cause adverse effects, the main ones being pain, fatigue, dyspnea, physical limitation, and weight loss, which can also significantly affect physical, emotional, social, and cognitive functions^4^ ^,^ ^8^ ^,^ ^9^.

Few studies have evaluated patient-related factors after surgical treatment of soft tissue sarcomas. Many include tumors of the extremities, as well as head and neck, trunk, and retroperitoneum sarcomas, making the results heterogeneous. 

Kask et al.^8^ applied the QLQ-30 questionnaire and other assessment instruments with an interval of six to 149 months after the main surgical treatment. The mean score was 75 (7-100), with 19% to 51% of the patients completely asymptomatic. For the QLQ-30, 10% of 137 patients were asymptomatic. Considering the multiple model, the QLQ-30 was impacted by age over 80 years, obesity, and need for reconstruction.

Heaver et al.^9^ demonstrated that advanced age at the time of sarcomas’ surgical treatment was associated with poorer quality of life. That study included patients with benign lesions and bone, hematological, and metastatic tumors. The authors justified the association between advanced age and poorer quality of life due to the vulnerability of older age and lower rehabilitation capacity in relation to younger people.

Female sex was also a determinant of worse postoperative functional outcome in the study by Saeybye et al.^10^. Sex impacts the quality of life after surgical treatment of sarcomas in those studies that include sarcomas of different parts of the body and locally aggressive benign lesions.

Silva et al.^11^ applied the EORTC QLQ 30 protocol and demonstrated similar quality of life in patients undergoing conservative surgeries and surgical amputation procedures. Constipation was more frequent in patients undergoing amputation. The authors attribute these findings to patients with more advanced tumors and greater use of opioids.

Improvement in quality of life is associated with the preservation of vascular and nerve structures during surgical procedures, which result in better functionality in rehabilitation programs in a multidisciplinary context, regardless of the surgical procedure offered, whether conservative surgery or amputation^11^ ^-^ ^14^.

The present study found a predominance of females compared with males, as did the study conducted by Lima et al.^14^, that considered female sex a factor for worse results in quality of life after surgical procedure.

Pain is often described as the most common symptom in oncology, with incidences that reach up to 90% of cases^15^. We observed pain as the most prevalent symptoms and with a frequency lower than that described in the literature. This is due to the period in which the approach was made with the QLQ-30 instrument, since the patients are in different postoperative follow-ups, and a percentage of them are undergoing chemotherapy treatment, outpatient follow-up being carried out in all visits by the general oncology and pain teams, constantly reviewing and managing the patients’ symptoms.

The prognostic value and practicality of using the American Society of Anesthesiologists (ASA) Physical Status Scale in oncology has already been evidenced by Zequi et al.^7^. None of the patients participating in the present study were classified as ECOG 3 or ASA 4, and these findings may be associated with a higher global QoL.

The EORTC QLQ-C30 questionnaire is widely recognized as one of the main instruments for assessing QoL in oncology. Its notability stems from its simple composition, containing only 30 items, making it easy to apply and understand. In addition, it is important to highlight that the questionnaire has been translated into Portuguese and validated in several studies, which increases its usefulness in Brazilian contexts^5^ ^,^ ^6^.

Fatigue is often identified as one of the most disabling and common symptoms in patients with advanced stage neoplasms^16^. Among the symptoms evaluated, fatigue was the symptom with the second highest average. Lima et al.^14^ observed fatigue as the main symptom, associating these findings with pre-existing clinical and psychological conditions. We believe that the significant average observed for fatigue is a result of the impact of both the patient’s disease and treatment, as demonstrated by their performance status.

Among the predominant symptoms in the study, we observed the highest averages for loss of appetite, insomnia, and constipation. Cancer can trigger malnutrition, resulting in loss of appetite. This effect can be attributed to the action of different mediators of inflammatory response, such as tumor necrosis factor, interleukins, and proinflammatory cytokines^17^. Chemotherapy itself can also have side effects in the gastrointestinal tract, which also contribute to the reduction of food intake.

Therefore, nutritional monitoring plays a fundamental role in the patient’s well-being and treatment continuity. It is essential that the medical professional is ready to prescribe appetite-stimulating medications, antiemetics, and enteral feeding options, thus ensuring that treatment is not interrupted. However, it is important to always keep the multidisciplinary approach continuous throughout the treatment of the malignant disease.

The highest mean observed in the EORTC QLQ-30 questionnaire was for the global measure of quality of life, with a value of 73.75. Lima et al.^14^ found the highest mean for the component of the global measure of quality of life, reaching 79.99. Bertoncello et al.^18^ highlighted a linear relationship between the various aspects of quality of life. They noted that when there is a high frequency of debilitating symptoms, it negatively impacts patients’ quality of life.

The high mean identified in this study is due to the low incidence of symptoms that restrict daily activities, such as pain and dyspnea. In addition, the low average recorded for the item related to financial difficulties, which exert an adverse impact on the quality of life of patients, stands out.

The present study has some limitations, such as the reduced sample, which is consistent with the literature, the cross-sectional nature, and the lack of analysis for an exclusive neoplastic type, since the questionnaire was applied at any time after surgery and to patients with a variety of soft tissue sarcomas.

## CONCLUSION

We consider this study to be significant, since our data corroborate those presented in the literature, highlighting the importance of rehabilitation for patients undergoing wide and three-dimensional tumor resection.
